# Dyslipidemia in Psoriasis: A Case Controlled Study

**DOI:** 10.1155/2014/729157

**Published:** 2014-10-08

**Authors:** Y. C. Nakhwa, R. Rashmi, K. H. Basavaraj

**Affiliations:** ^1^Department of Dermatology, Mahatma Gandhi Medical College & Research Institute, Pondicherry 605007, India; ^2^Department of Dermatology, JSS Medical College, Mysore 570015, India

## Abstract

Multiple observational studies have demonstrated associations of psoriasis with metabolic syndrome including obesity, diabetes, hypertension, dyslipidemia, and osteoporosis. However there is paucity of Indian studies on dyslipidemia in psoriasis. The aim of this study was to assess the serum lipids in psoriasis and to investigate the association of lipids with disease severity and its duration. 100 cases of psoriasis (75/M, 25/F), between 15 and 72 years, were recruited with age and sex matched 73 controls. Using Psoriasis Area Severity Index (PASI) cases were graded into mild, moderate, and severe psoriasis. Serum total cholesterol and triglycerides were analyzed using enzymatic method. Using independent *t*-test, significant elevation of serum cholesterol, triglycerides, high density lipoprotein (HDL) and very low density lipoprotein was observed (*P* < 0.05) when compared to controls. The levels of low density lipoproteins were comparable in cases and controls. Lipid aberrations in hypertensive patients were significant. There was a decrease in HDL levels with increase in disease severity. A fall in the levels of HDL was seen in cases with long term psoriasis. There is a strong association of dyslipidemia with psoriasis. There exist racial and ethnic variation in the prevalence of psoriasis; however, dyslipidemia is consistently seen in diverse population. Whether genetic factors are implicated in lipid derangements in psoriasis needs further research.

## 1. Introduction

Psoriasis is a chronic immunologically based inflammatory disease of skin. It has been estimated to affect around 2% of the population [1]. Various studies have demonstrated the association of psoriasis with increased oxidative stress and decreased antioxidant capacity. Time and again multiple studies have observed the occurrence of lipid aberrations, hypertension, diabetes, obesity, and now osteoporosis in psoriasis patients [2]. Are these changes coincidental? The metabolic syndrome—which comprises abdominal obesity, arterial hypertension, abnormal oral glucose tolerance, and abnormal blood lipids—is the most important comorbidity of psoriasis [3]. Psoriasis and its comorbidities, including obesity, contribute to a patient's systemic inflammatory burden which in turn causes insulin resistance [4]. This results in a state in which the equilibrium between proatherogenic and antiatherogenic effects of insulin is shifted towards proatherogenic effects [4]. Though literature suggests a definitive association of lipid derangements with psoriasis, the available data is contradictory and fails to provide a definite conclusion [5–10]. However to the best of our knowledge, study on dyslipidemia in psoriasis in Indian population is lacking. Hence this study was undertaken to find what happens to lipid profile in Indians with psoriasis and observe the variation of lipid levels with disease severity and disease duration.

## 2. Material and Methods

### 2.1. Cases

Cases included psoriasis patients older than 15 years who came to the Outpatient Department of JSS Medical College and Hospital from October, 2007, to July, 2009. A total of 100 consecutive cases who met the inclusion criteria were enrolled. Measurement of skin disease severity was performed using Psoriasis Area Severity Index (PASI). A PASI score below 3 was defined as “mild,” between 3 and 10 was defined as “moderate,” and above 10 was defined as “severe” disease (according to British Association of Dermatologists Guidelines) [10]. Exclusion criteria were pustular psoriasis, erythrodermic psoriasis, diabetes, obesity, family history of hyperlipidemia, renal and liver failure, and patients on retinoids or lipid lowering agents.

Study was approved by Ethics Committee of JSS Hospital. A written consent was obtained from patients/guardians.

### 2.2. Controls

Controls were selected by taking age and sex into consideration. Controls with past or ongoing medications that may affect lipid metabolism were excluded. 73 age and sex matched controls were recruited. Controls were thoroughly examined for any clinical evidence of psoriasis.

### 2.3. Questionnaire

Detailed history was obtained from cases and controls with regard to family history, prior treatment history, physical activity, and other lifestyle factors including tobacco and alcohol exposure. Data also included weight, height, body mass index (BMI), blood pressure, duration of psoriasis, type and severity of psoriasis, and presence of psoriatic arthropathy. BMI was calculated as weight (kg)/[height (mt)]^2^.

### 2.4. Blood Sampling

Venous blood was obtained after 12 hours of fasting. Blood was drawn into sterile vacutainers and centrifuged and serum was obtained.

### 2.5. Serum Lipid Analysis

Serum total cholesterol was measured by enzymatic end point cholesterol oxidase method. Serum total triglycerides (TGs) were estimated using enzymatic method—glycerol phosphate oxidase: peroxidase (GPO:PAP) method. High density lipoprotein (HDL) was measured using direct method. Very low density lipoprotein- (VLDL-) cholesterol was estimated using formula: VLDL-cholesterol = TGs/5. Serum low density lipoprotein (LDL) was estimated using Friedwald's formula [11]:

(1) LDL-cholesterol=  Total  Cholesterol−(VLDL-cholesterol+HDL-cholesterol).

### 2.6. Statistical Analysis

All the statistical methods were carried out through SPSS for Windows (version 16.0).

The prevalence of psoriasis according to age and sex was calculated and compared with the controls using cross-tabulation procedure. The statistical significance was calculated by one-way analysis of variance (ANOVA) and significance level used was at 95% confidence level.

The mean values of serum lipids in cases and controls were compared using independent samples *t*-test. One-way ANOVA was used to study the variations in serum lipid levels based on type of psoriasis, its duration, and severity. To account for the effect of confounding factors like smoking, alcoholism, and hypertension multivariate logistic regression was performed using chi-square test.

## 3. Results

In this case control study of 100 cases and 73 controls the age range of the cases was 15–72 years. The descriptive characteristics of study population are given in Table 1. Student's *t*-test did not show significant statistical difference in age and sex of cases and controls.

Cases had mild to severe psoriasis with PASI ranging from 0.8 to 50.8 with a median 6.9. 19% of the cases had PASI < 3, 45% had PASI between 3 and 10, and 36% had PASI > 10.

The most common clinical type of psoriasis was chronic plaque variety accounting for 79% of cases. Out of 79 cases, 48 had associated scalp involvement. Another 5% of cases showed localized scalp psoriasis. Thus scalp involvement was seen in 53% of the patients. Palmoplantar psoriasis was seen in 10% of cases. 3 out of 100 cases showed flexural involvement also known as “inverse psoriasis.” Psoriatic arthropathy was seen in 3 cases.

The serum total cholesterol was significantly elevated in cases when compared to controls. The mean serum total cholesterol was 189.31 ± 37.84 mg/dL in cases, whereas in controls it was 170.79 ± 36.25 mg/dL (*P* = 0.001). Mean HDL-cholesterol levels in cases were 43.01 ± 7.97 mg/dL which were higher when compared to the mean in controls (37.47 ± 12.18) mg/dL. The elevation was significant (*P* = 0.001) in cases.

**Figure 1:**
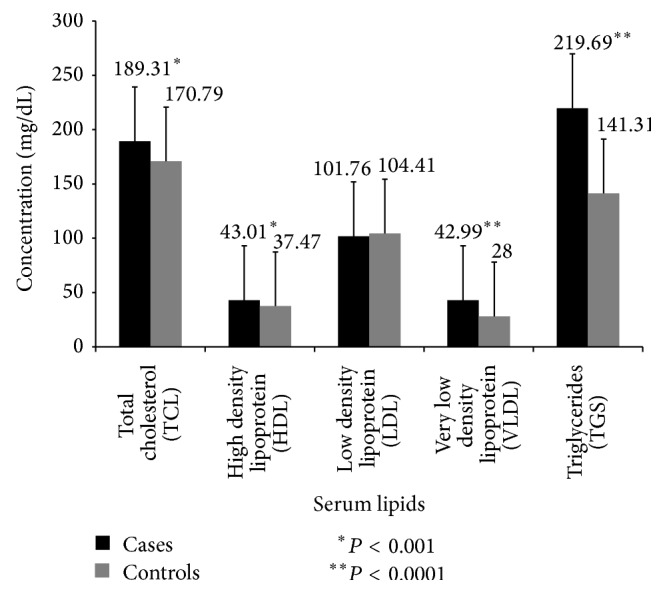
Comparison of concentration of serum lipids in cases and controls. The mean of serum cholesterol, high density lipoprotein, very low density lipoprotein, and triglycerides in cases was significantly elevated. Results are expressed as mean ± S.D. ^*^ *P* < 0.05 is considered to be statistically significant.

Serum triglyceride levels were analyzed. In cases, the mean was 219.69 ± 105.46 mg/dL which was significantly higher than controls. Elevation of VLDL in cases was noted (mean 42.99 ± 20.52). The mean in controls was 28.0 ± 11.47 mg/dL. The difference was significant at *P* = 0.0001. No difference in LDL was found in cases as compared to controls (Figure 1).

Out of 100 cases, 7 patients had hypertension. (Systolic BP > 140 mmHg, diastolic > 90 mmHg.) The group statistics are described in Table 2. The difference was statistically significant for total cholesterol, VLDL, and TGs. In hypertensive cases the mean HDL was lower than the group mean, but the difference was not significant.

**Figure 2:**
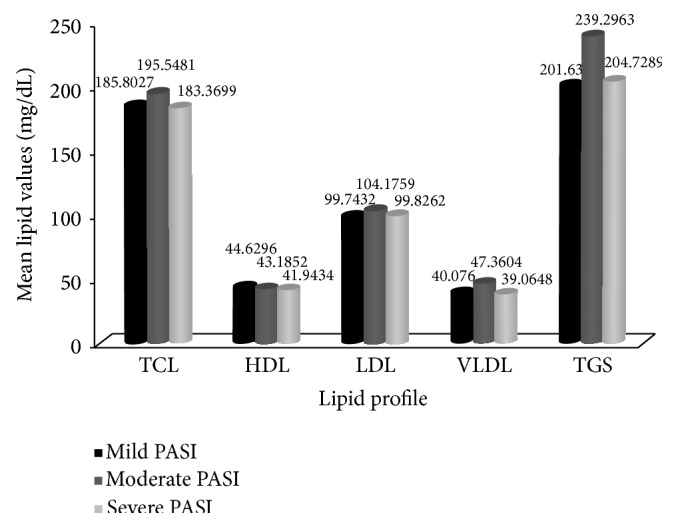
Variations of mean serum lipoproteins in psoriasis patients based on disease severity. Results are expressed as mean + S.D. *P* < 0.05 is considered to be statistically significant.

Variations in serum lipids were observed with disease severity. No specific trend was noticed except in HDL-cholesterol. The levels decreased with increasing severity, but the difference was not statistically significant (*P* > 0.05) (Figure 2).

**Figure 3:**
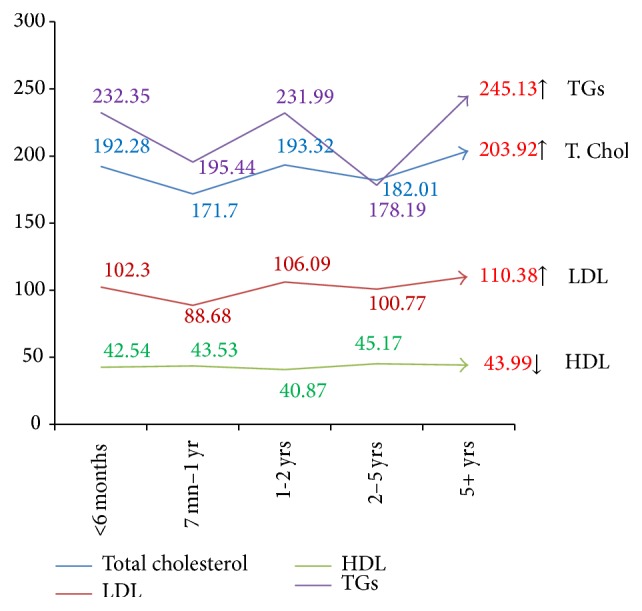
Variations of mean serum lipoproteins in psoriasis patients with duration of disease. Results are expressed as mean + S.D. *P* < 0.05 is considered to be statistically significant.

Lipid levels were assessed taking disease duration into consideration. The comparison of total cholesterol, LDL, HDL, and TGs and their variation with disease duration is depicted in Figure 3.

No significant variations in lipid levels were observed in different types of psoriasis. 3 cases that had inverse psoriasis did not show any significant change. 23 cases were chronic smokers and 10 cases were alcoholics. Through multivariate logistic regression, the effect of various confounding factors like smoking, alcoholism, and obesity was found to be nonsignificant through chi-square test.

## 4. Discussion

Half a century ago Lea Jr. et al. [6] reported increased serum lipid levels in psoriasis patients. Later much research was carried out to find the concentration of lipids in scales of psoriasis patients [12]. Subsequently multiple observational studies were carried out in heterogeneous study population consistently pointing towards dyslipidemia. Aberrant lipoprotein composition at disease onset observed in a study indicates predisposition of psoriasis patients to develop lipid abnormalities [7].

Various studies done in Caucasians have reported increased [8, 9] as well as normal [13] serum total cholesterol. Serum triglyceride levels have shown a significant elevation in most studies [9, 13]. Numerous studies [8, 13, 14] have reported normal levels of serum HDL, whereas Rocha-Pereira et al. [9] reported a fall. However most studies report significant elevation of the most atherogenic LDL [8, 9, 13, 14] in psoriasis.

Thus various studies reported contradictory results. In our study significant elevation of serum total cholesterol, TGs, VLDL, and HDL was observed, whereas LDL levels were comparable in cases and controls. Thus we report elevation of protective HDL and normal levels of most atherogenic lipid LDL which is not consistent with the above mentioned reports.

PASI score was used to grade the cases into mild, moderate, and severe. This score is considered as outstanding when severe cases are involved. It also provides the advantage of a large base of studies in which it has been used for comparison [15]. We considered PASI as we had included large number of severe cases. There was no significant correlation of dyslipidemia with disease severity based on PASI; however, we observed a fall in the levels of protective HDL in severe psoriasis as compared to mild cases.

In our study, there was an increase in the serum total cholesterol levels and LDL, VLDL, and triglyceride levels but a fall in the levels of HDL in patients with long term psoriasis (more than 5 years). Though the changes in HDL based on disease severity and duration were not significant, they form an interesting observation. HDL levels, which are protective against cardiovascular risk, show changes different from other lipids. This could signify increased risk and systemic damage with disease severity and duration.

Till date all accumulated knowledge is derived from studying psoriasis patients without considering disease duration. Mallbris et al. [7] showed the lipid changes at the onset of the disease. What happens to these lipid levels as the disease progresses is yet to be evaluated. According to our study, an increasing trend in the lipids was observed with the disease duration. Whether this change indicates the increasing cardiovascular risk with the progression of psoriasis needs long term followup of these cases and corroboration with larger case population.

In the study conducted by Mallbris et al. [7] on 200 patients lipid levels were studied taking environmental risk factors into consideration. They found that factors like smoking, high alcohol consumption, and low physical exercise were not the likely explanation for lipid abnormalities. Our statistical analysis proved that dyslipidemia was associated with psoriasis even after controlling for confounders like smoking and alcohol intake.

Patients with hypertension had significant elevation of lipid abnormalities than others. However the number of patients with hypertension in our study was less (7%). Hypertension along with dyslipidemia adds to the comorbidities of psoriasis. These together cause increase in the systemic inflammatory burden. There was a definite increase in serum lipids in hypertensive psoriatic patients when compared to nonhypertensive cases and controls in our study which might suggest an accelerated “psoriatic march” causing an early development of insulin resistance [4]. Whether hypertension acts synergistically with psoriasis to produce aberrations in lipid profile needs to be proved beyond doubt.

Various external and internal factors may possibly work together leading to lipid aberrations in psoriasis. The chronic nature of the disease influences lifestyle of the patient setting up a vicious cycle. Smoking, increased alcohol intake, and stress increase the oxidative damage in the body [16]. Obesity is an important comorbidity reported in psoriasis which also contributes to the cardiovascular morbidity [5, 16].

Psoriasis is a T helper cell 1 response, leading to increased levels of TNF-*α* [3] which are also found in the atherosclerotic plaque contributing to cardiovascular morbidity [16]. TNF-*α* is also shown to cause insulin resistance [5] which is suggested to interfere negatively with lipid metabolism. Changes in TNF-*α* levels with the duration of psoriasis could provide more definitive answer to hyperlipidemia in patients with long term psoriasis.

The lipids present in the scales of psoriasis have shown increased levels of cholesterol and low free fatty acids [12]. During exfoliation there is loss of cholesterol from the scales. This could be the reason for increased synthesis of serum cholesterol causing dyslipidemia. Functional and structural abnormalities have been reportedly seen in various segments of gastrointestinal tract [8]. Intestines play an important role in the absorption, composition, and degradation of lipoproteins. Thus there is a possibility that the structural abnormalities in the gastrointestinal tract can adversely affect the lipid levels.

We are well aware of the following limitations in our study. First diabetes in the patients was ruled out based on history and no diagnostic test could be performed. Second, no detailed history regarding smoking and alcohol intake could be obtained from the controls. Third, we could not perform followup lipid analysis in these patients. The numbers of hypertensive psoriatic cases were few.

## 5. Conclusion

We could establish that the occurrence of dyslipidemia in psoriasis is not just coincidental but there is a definite association. Our study shows no significant correlation of lipids with disease severity and duration. This strengthens the fact that psoriasis is a systemic disease. We can conclude that severe psoriasis cases should be screened for lipid abnormalities which can help in early detection of lipid dysfunction and cardiovascular comorbidity.

## Figures and Tables

**Table 1:** Demographic characteristics of cases and controls.

	Cases	Controls	*P* value
Males-*N*	75	55	
Females-*N*	25	18	
Age range	15–72 yrs	18–73 yrs	
Mean age	40.72 ± 13.4	41.67 ± 11	0.620

**Table 2:** Comparison of serum lipid levels in controls and nonhypertensive and hypertensive cases with psoriasis.

	Controls (*n* = 73)	No hypertension (*n* = 93)	Hypertension (*n* = 7)	*P* value
Total cholesterol	170.79 ± 36.25	187.03 ± 35.94	219.57 ± 51.7	0.028^*^
HDL	37.47 ± 12.18	43.11 ± 8.17	41.71 ± 4.53	0.657
LDL	104.41 ± 26.51	100.83 ± 26.41	114.14 ± 27.47	0.203
VLDL	28.00 ± 11.47	41.45 ± 18.64	63.42 ± 33.15	0.006^*^
TGs	141.31 ± 57.90	212.29 ± 96.86	318.0 ± 165.91	0.010^*^

^*^ *P* value < 0.05.
